# Effect of bilateral paravertebral nerve block on cognitive function in elderly patients undergoing radical gastrectomy for gastric cancer: a prospective randomized double-blind controlled trial

**DOI:** 10.1186/s12871-022-01764-9

**Published:** 2022-07-15

**Authors:** Yanhong Shang, Fuwei Qi, Zhong Zheng, Guangyu Yang, Fan Fei, Qiang Guo, Kangle Zhu

**Affiliations:** 1 Department of Anesthesiology, The First People’s Hospital of Taicang, Taicang Affiliated Hospital of Soochow Univercity, Jiangsu Province 215413 Suzhou City, China; 2 Department of Medicine, Xinglin College, Nantong University, Nantong City, 226007 Jiangsu Province China

**Keywords:** Cognitive dysfunction, The elderly, Paravertebral nerve block, Gastric cancer

## Abstract

**Objective:**

To investigate the effect of a bilateral paravertebral block (PVB) on cognitive function in elderly patients undergoing radical gastrectomy for gastric cancer.

**Methods:**

Sixty patients (40 men and 20 women) aged 65–80 undergoing radical gastrectomy surgery under general anaesthesia were included and randomly assigned to either the PVB group or the control group. Patients in the PVB group had before incision a single-shot ultrasound-guided bilateral PVB at the T8 level with 20 mL of ropivacaine 0.375%, while patients in the control group had no block. Patients in both groups had a BIS-guided total intravenous anaesthesia with propofol and remifentanil infusions. Postoperative cognitive function assessed by the mini-mental state examination (MMSE) and NSE (neuron-specific enolase) was the primary outcome.

**Results:**

The awareness time in group PVB was shorter than that in the group C, and the propofol and remifentanil dosages were less than that in group C (*P<*0.001*, P* = 0.007, respectively). Furthermore, the change of the MMSE score and the NSE concentration was significant from day0 to day1 and day1 to day2. (*P<*0.001).

**Conclusion:**

A single-shot bilateral PVB active throughout radical gastrectomy for gastric cancer reduces the needs for general anaesthetic agents and improve postoperative recovery, along with a surrogate evidence for neuroprotective effects.

**Trial registration:**

ChiCTR2200060088. Registered 18 May 2022.

## Introduction

Gastric cancer (GC) is the most common malignant tumor in the digestive tract in China, which brings a huge disease burden to society. Elderly patients are the high-risk group for the development of GC [1], and surgery is an important means to treat early GC. Most of patients developed perioperative complications, such as postoperative pain, hypothermia and central nervous system complications. Postoperative cognitive dysfunction (POCD) is one of the common complications of the central nervous system after surgery and anesthesia, which is mainly manifested as postoperative memory, abstract thinking, disorientation, and decreased social activity ability [2]. POCD may be related to the surgical site, anesthesia method, and postoperative pain [3]. The incidence of POCD in elderly patients undergoing abdominal surgery is as high as 60%, resulting in increased hospital stay, medical expenses, and medical resource consumption. It is urgent to find approach to prevent the occurrence of POCD.

Paravertebal block (PVB), also named as paravertebral nerve block (PVNB), is a nerve block achieved by injecting anesthetics at the outlet of the nerve root in the intervertebral foramen (thoracic paravertebral space), which can produce ipsilateral somatomotor, sensory, and sympathetic nerve blocks, mainly used for regional anesthesia and postoperative analgesia in the chest and abdomen [4]. Hugo Sellheim et al. first used paravertebral nerve block for abdominal analgesia in 1905. After decades of development, paravertebral nerve block has surpassed continuous epidural anesthesia for analgesia and is widely used for perioperative analgesia in thoracotomy. At present, TPVB has been widely used for perioperative analgesias such as rib fracture, pleural effusion, open cholecystectomy, hepatobiliary surgery, inguinal hernia repair, breast cancer surgery, and thoracotomy [5]. Wei et al. compared different postoperative analgesic strategies on postoperative neurocognitive function of lung cancer patients and found that thoracic paravertebral block could protect postoperative neurocognitive function of patients. Wang [6] et al. showed that ultrasound-guided bilateral paravertebral nerve blocks had a significant clinical effect and good analgesic effect for radical gastrectomy. However, the effect of PVB on the postoperative cognitive function of patients with GC is still unclear. This study was designed to explore the effects of bilateral paravertebral nerve block combined with general anesthesia on the postoperative cognitive function of elderly patients with radical gastrectomy.

## Materials and methods

### Study design

The study was a prospective randomized double-blind controlled trial. All methods were carried out in accordance with relevant guidelines and regulations or Declaration of Helsinki. This study was approved by the Ethics Committee of the First People’s Hospital of Taicang and the informed consent forms were signed for all enrolled patients. Our study was registered with Trial registration: ChiCTR2200060088.

### Study participant

**Fig. 1:**
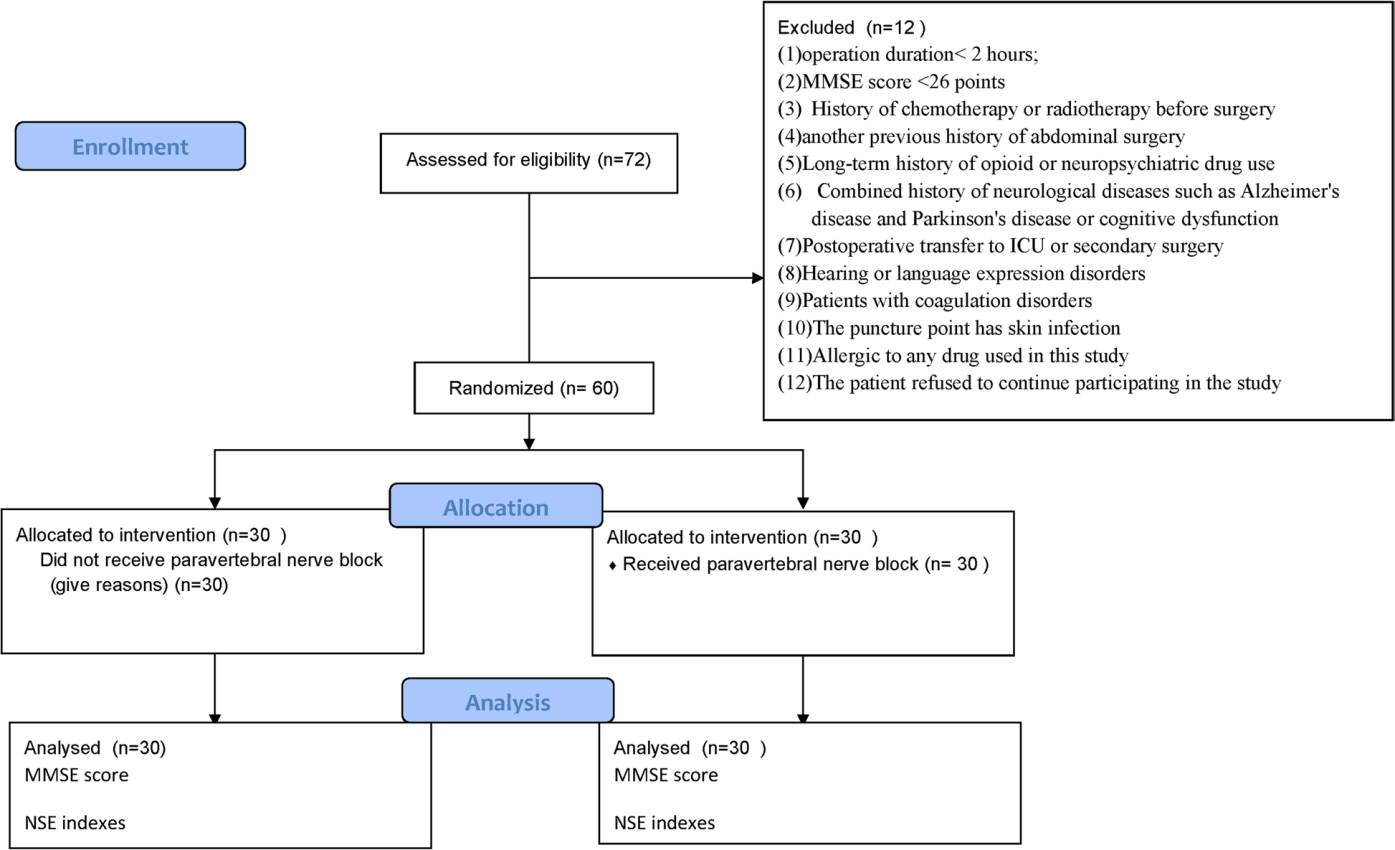
Research flow chart

Patients undergoing elective gastrointestinal tumor surgery in our hospital from January 2019 to December 2021 were included in the study. Inclusion criteria: Patients of any gender, 65 ~ 80 years old, ASA grades II ~ III, and retrograde elective gastrointestinal surgery. Exclusion criteria: (1) duration of operation< 2 hours; (2) MMSE (mini-mental state examination) score < 26 points (3) History of chemotherapy or radiotherapy before surgery (4) another previous history of abdominal surgery (5)Long-term history of opioid or neuropsychiatric drug use (6) Combined history of neurological diseases such as Alzheimer’s disease and Parkinson’s disease or cognitive dysfunction (7) Postoperative transfer to ICU or secondary surgery (8) Hearing or language expression disorders (9) Patients with coagulation disorders (10) The puncture point has skin infection (11) Allergic to any drug used in this study (12) The patient refused to continue participating in the study, as shown in Fig. 1.

Sixty patients were enrolled and randomly divided into two groups according to the random number table: the control group (Group C, 30 cases) and the paravertebral nerve block group (PVB group, 30 cases). The anesthesiologists who performed anesthesia management and the researchers who followed up after the operation were blinded. Anesthesia management and nerve block procedures in this study were performed by experienced anesthesiologists.

All patients included in the study did not receive preoperative medication, and monitoring was established after the patient entered the room: percutaneous oxygen saturation (SpO2), non-invasive blood pressure monitoring, electrocardiogram, and Bispectral Index (BIS). After the peripheral venous access was opened, lidocaine local anesthesia was applied for radial artery puncture catheterization and invasive arterial blood pressure was monitored. After intravenous injection of 1 mg midazolam and 5 μg sufentanil citrate in the PVB group, the patient was asked to lie on his/her side. After routine disinfection and drape laying, he/she was scanned and positioned along the spine by Sonite ultrasound apparatus so that the direction of the convex array probe was parallel to the ribs and positioned to the T8 vertebral body. The thoracic para-space was identified in the ultrasound image, and the needle was inserted into the plane. After the puncture needle broke through the costal transverse process ligament and drew back the airless, blood, and cerebrospinal fluid, 20 ml of 0.375% ropivacaine was injected, and the sliding pleural downforce was visible. Complete the other side of the T8 paravertebral block in the same way. The patients were observed for 20 min after the block and the block plane was tested by acupuncture. If there was no obvious block plane, the block was considered a failure. Group C has no special treatment.

### Anesthesia methods and postoperative analgesia

All patients received intravenous anesthesia during the operation. Midazolam 0.04 mg/kg, etomidate 0.3 mg/kg, sufentanil citrate 0.5 μg/kg and atracurium cis-benzenesulfonate 0.2 mg/kg were used for anesthesia induction. After induction, intubation was performed orally under the guidance of a visual laryngoscope and connected to the anesthesia machine for mechanical ventilation. The endotracheal tube was fixed. The lower part of the head was taken, deep venipuncture was performed through the right internal jugular vein of the patient, and catheterization was performed. During the operation, the pulmonary protective ventilation strategy was adopted: tidal volume of 6–8 mL/kg, the inhaled oxygen concentration of FIO2 50–80%, PEEP 4–8 mmH20, and suction to shout ratio of 1: 2. The respiratory frequency was adjusted to 12–15 times/min to maintain the end-tidal carbon dioxide (PetCO2) 35 ~ 40 mmHg during the operation. The anesthetics were maintained by continuous intravenous infusion of propofol 0.08–10 ~ 15 μg kg ^− 1^ min ^− 1^, atracurium besylate 0.05–0.1 mg kg ^− 1^ h ^− 1^, and remifentanil 0.15–0.3 μg kg ^− 1^ min ^− 1^, with intermittent addition of sufentanil citrate 10–15 μg. During the operation, medication dosage was adjusted according to the monitored values of patients’ BIS values to maintain the fluctuation of BIS values within the range of 40–60. The intraoperative blood pressure management goal of the patient was to give appropriate fluid replacement and vasoactive drugs such as phenylephrine and ephedrine within the range of 20% above and below the preoperative basic blood pressure to achieve the target value.

Atracurium besylate was stopped 20 min before the end of the operation. Propofol was stopped when the surgeon sewed the skin. Remifentanil and Parecoxib 40 mg were added after the operation. The patient was transferred to PACU with a catheter. Patient-controlled intravenous analgesia (PCIA) pump was used in all postoperative analgesia programs, and the prescription was as follows: 100 μ g of sufentanil citrate+ 10 mg of tropisetron hydrochloride; normal saline was added for dilution to 100 ml; no background dose was given; bolus dose was 2 ml, and the locking time was 15 min. Neuromuscular blockade was not reversed with reversal agents in all patients during the awakening period after the operation until the patients were fully awake and able to respond to calls, the throat reflex, swallowing reflex and cough reflex had fully recovered, and the tidal volume and minute ventilation volume had returned to normal. All patients were observed in the PACU for 1 h after extubation, and no special cases could be transferred to the ward.

### Observation indicators

The patients in the two groups received a mini-mental state examination (MMSE) 1 day before surgery (D0), 1 day after surgery (D1), and 3 days after surgery (D2), which included five aspects: directional power, memory, language power, computational power, and directional power. The total score was 30 points. The higher the score was, the stronger the cognitive function would be. The peripheral venous blood was collected from the patients at time points D0, D1, and D2, and centrifuged at 3000 r/min. The serum was collected and stored in cold storage. and the enzyme-linked immunosorbent assay (ELISA) was used to determine NSE (neuron-specific enolase) and detect the concentration of neuron-specific enolase, NSE. The level of human NSE was measured by double antibody sandwich method. The absorbance (OD value) was measured with an enzyme labeling instrument at the wavelength of 450 nm, and the concentration of NSE in the sample was calculated through the standard curve.

Record the operation and anesthesia-related information: duration of operation, anesthesia duration, extubation duration, type and the total amount of infusion liquid during the operation, intraoperative bleeding and urine volume, and the total dosage of propofol and opioids during the operation.

### Statistical methods

The sample size was calculated according to the results of the preliminary experiment, whether PVB was applied to the MMSE score of the two groups 24 h after surgery or not. Setting the test level α = 0.05, 1-β = 0.8, the sample size was calculated by STATA 10.1 for 50 cases. The shedding rate was set to no more than 20%. The final calculation was 30 cases in each group with the sample size of 60 cases.

All data were statistically analyzed by SPSS 15.0 (IBM Corp., Armonk, New York, USA). Kolmogorov–Smirnov-test was used to assess normal distribution, t-test was used for inter-group comparison. All non-normally distributed variables as median (1 st quartile – 3 rd quartile) and compared using the nonparametric *Mann*-*Whitney test*. Enumeration data were expressed as a rate (%) and inter-group comparisons were performed by the chi-square test or Fisher’s exact probability method. Type I error inflation was controlled with a Bonferroni-Holm correction. *P* < 0.05 indicated that the difference was statistically significant.

## Results

### General information

There was no significant difference in age, gender, BMI, ASA stage, years of education, basic disease, and preoperative MMSE score between the two groups (Table 1).

**Table 1:** General data and operation-related information of patients in the two groups (median [1st quartile – 3rd quartile] or n/%)

	C group(*n* = 30)	PVB group(*n* = 30)	*P*
Age (years)	65.5(64.5–75)	66(65–78)	0.374
Gender (male/%)	22/73.3	18/60.0	0.273
BMI (kg/m2)	24.5(22.5–26.2)	24.60(21.5–26.2)	0683
Years of education	5.3 (3.0–7.0)	6.0(4.0–7.0)	0.431
ASA staging (II/III)	20/10	18/12	0.592
Hypertension (case/%)	21/70.0	19/63.3	0.584
Diabetes (Cases/%)	18/60.0	15/50.0	0.436

e: Compared with group C, * *p* < 0.05. Data: median [1st quartile – 3rd quartile] or n%

### Patients in two groups: operation and anesthesia related information

Nerve block procedures in the PVB group were successful and there were no complications. The differences in operation time and total anesthesia time were not significant among groups (*P* > 0.05). The time from the end of surgery to tracheal extubation in PVB group was shorter than that in group C (*P* < 0.05). The doses of propofol and remifentanil in PVB group were lower than those in group C (*P* < 0.05) (Table 2).

**Table 2:** Intraoperative situation of two groups of patients (median [1st quartile – 3rd quartile])

	C group(*n* = 30)	PVB group(*n* = 30)	*P* value
Duration of operation (min)	197(162–231)	177(163–221)	0.550
Time to extubation (min)	9.00(9.00–10.00	7.00(6.00–8.00)	< 0.0001
Propofol (mg)	1235.00(972.50–1325.00)	917.50(820.00–1157.00)	< 0.0001
Remifentanil (μg)	2.35(1.70–3.275)	1.750(1.20–2.60)	0.0070

Note: Compared with group C, * *p* < 0.05. Data: median [1st quartile – 3rd quartile]

### Comparison of MMSE and NSE levels during the perioperative period between the two groups

Next, we compared the change value of MMSE scores and NSE indexes from D0 to D1 and D0 to D1 of two groups. As showed in Table 3, the change of MMSE scores and NSE indexes in two period showed significant difference in two groups (*P* < 0.05).

**Table 3:** Comparison of MMSE and NSE levels between the two groups during the perioperative period

	C group	PVB group	*P* value
NSE (μg/L)			
[%]change in D1	106.0(73.3–182.9)	63.5(39.1–100.6)	< 0.0001
[%] change in D2	32.5(11.3- 63.5)	13.06(2.4–43.5)	0.041
MMSE (score)			
[%]change in D1	14.3(0.8- 18.4)	5.6(3.7- 10.3)	< 0.0001
[%]change in D2	7.4(4.2- 9.9)	1.5(0.1- 5.0)	< 0.0001

Note: Compared with group C, * *p* < 0.05. Data: median [1st quartile – 3rd quartile]

## Discussion

Herein, we found that the patients in PVB group had the shortened duration of extubation and consumed less remifentanil and propofol than the group C. Furthermore, the change of MMSE scores and NSE indexes in PVB group was lower than those in the group C. All data suggested that preoperative application of ultrasound-guided bilateral paravertebral nerve blocks can reduce intraoperative propofol and remifentanil dosage, postoperative awareness time and reduced the POCD.

The symptoms of POCD range from mild memory loss to the inability to concentrate or process information received by the brain, thus severely affecting the quality of life of patients, especially elderly patients [7]. MMSE is widely used in cognitive function screening, mainly for simple evaluation of orientation, memory, language, computation,and attention, etc. In this study, the changes of MMSE scores of the patients in the PVB group were significantly than those in group C, suggesting that preoperative implementation of PVB could reduce the use of anesthetics in the surgery of patients and reduce the degree of postoperative nervous system damage. That nerve block during surgery can improve the postoperative cognitive function of elderly patients [8], which is similar to the conclusion of this study.

NSE is an important serological indicator reflecting postoperative cognitive dysfunction, and its activity is highest in brain cells. The change of activity of NSE is closely related to many neurological diseases caused by nerve injury. The increase of serum NSE concentration can sensitively reflect neuronal injury. In the circumstance of neuron damage, large amounts of NSE leak from the damaged neurons rapidly and enter the brain spinal fluid and systemic circulation through the damaged blood-cerebrospinal fluid barrier. Elevated levels of NSE in blood and cerebrospinal fluid are consistent with the extent of neuronal damage. Therefore, the determination of NSE content can reflect the degree of brain injury and is a biochemical indicator reflecting the specificity of central nervous system injury. A recent study [9] showed that the NSE of elderly patients increased at 12 h and 1d after surgery, and the peak appeared at 12 h after surgery, indicating that elderly patients have different degrees of brain injury after surgery, and the measurement of NSE within 24 h is the best. The changes of NSE in the PVB group was significantly higher at 12 h, 1 d, and 2 d after surgery t indicating that PVB may occur if NSE has not recovered to the preoperative level 1 d after surgery. Thus we measured the NSE 2 days postoperatively. The outcome showed that the change of serum NSE in the PVB group was significantly lower than that in the group C on day 1 and day 3 after surgery, suggesting lessening postoperative cognitive dysfunction.

Paravertebral nerve block refers to the simultaneous block of somatosensory and motor nerves and analgesic effect achieved by injecting local anesthetics into the D1-D2 paravertebral space so that the lateral peripheral nerves of the spinal cord are surrounded by the local anesthetics. It is proposed in the guidelines for rapid rehabilitation of elderly patients that multimodal analgesia can reduce the perioperative use of opioids and accelerate the rehabilitation of patients [10]. Studies have confirmed that local anesthetics penetrate into other spaces through the vertebral body or epidural space connected with the injection points, so as to achieve the effect of blocking multiple segments. The failure rate of PVB technology under ultrasound guidance is only 2.9%, which can bring about comparable analgesic effects of epidural anesthesia. At the same time, the failure rate and complications of PVB technology are much lower than those of thoracic epidural block, thus improving the success rate of operation and reducing the complications [11]. Due to the special anatomical location of the paravertebral nerve block, it is mainly used in chest surgery and upper abdominal surgery, and the application of paravertebral nerve block in radical surgery for GC has certain feasibility. In this study, PVB procedures were successful and uncomplicated in all patients. In this study, the anesthetics used in the PVB group were all less than those in the C group, which might be at least partially responsible for the less cognitive dysfunction in PVB group [12]. The application of paravertebral nerve block before the start of surgery can reduce the use of anesthesia drugs during the operation for patients undergoing radical gastrectomy, and accelerate the patient’s postoperative recovery.

### Limitation

There are still many limitations in this study, and the conclusions of this study need caution in the clinical promotion and still need to be further validated by high-quality studies with clinical samples. Furthermore, as propofol and remifentanil are short acting, and as PVB reduced NSE at D1, this suggested that PVB might have a neuroprotective indirect effect. Therefore, haemodynamic data shall be considered. We did not assess postoperative pain, while PVB could also have protective effects on this outcome [13]. Additionally, further neuropsychological tests also should be considered.

## Conclusion

In summary, the current study showed that a single-shot bilateral PVB initiated before incision, and therefore active throughout the surgery of radical gastrectomy for gastric cancer, was able to reduce the needs for general anaesthetic agents, and to improve postoperative cognition and recovery. A neuroprotective action is suspected because of the positive effects on the biomarker NSE, but how the PVB could be neuroprotective remains a matter of debate, especially because the haemodynamic aspects were not assessed here.

## Data Availability

Due to concealment involving participants, privately anonymous datasets will be sent to by reasonable request corresponding author.

## Abbreviations

ASA: American Society of Anesthesiologists
BMI: Body mass index
PACU: Postanesthesia care unit
